# Assessing impacts of unconventional natural gas extraction on microbial communities in headwater stream ecosystems in Northwestern Pennsylvania

**DOI:** 10.3389/fmicb.2014.00522

**Published:** 2014-11-04

**Authors:** Ryan Trexler, Caroline Solomon, Colin J. Brislawn, Justin R. Wright, Abigail Rosenberger, Erin E. McClure, Alyssa M. Grube, Mark P. Peterson, Mehdi Keddache, Olivia U. Mason, Terry C. Hazen, Christopher J. Grant, Regina Lamendella

**Affiliations:** ^1^Juniata College, Department of BiologyHuntingdon, PA, USA; ^2^Department of Biology and Huck Institutes of Life Sciences, Pennsylvania State UniversityUniversity Park, PA, USA; ^3^DNA Sequencing and Genotyping Facility, Cincinnati Children's Hospital Medical CenterCincinnati, OH, USA; ^4^Department of Earth, Ocean, and Atmospheric Science, Florida State UniversityTallahassee, FL, USA; ^5^Department of Civil and Environmental Engineering, University of Tennessee KnoxvilleKnoxville, TN, USA; ^6^Biosciences Division, Oak Ridge National LaboratoryOak Ridge, TN, USA

**Keywords:** marcellus shale, fracking, 16S rRNA gene sequencing, next generation sequencing, methanotrophs, beta diversity, acidophilic

## Abstract

Hydraulic fracturing and horizontal drilling have increased dramatically in Pennsylvania Marcellus shale formations, however the potential for major environmental impacts are still incompletely understood. High-throughput sequencing of the 16S rRNA gene was performed to characterize the microbial community structure of water, sediment, bryophyte, and biofilm samples from 26 headwater stream sites in northwestern Pennsylvania with different histories of fracking activity within Marcellus shale formations. Further, we describe the relationship between microbial community structure and environmental parameters measured. Approximately 3.2 million 16S rRNA gene sequences were retrieved from a total of 58 samples. Microbial community analyses showed significant reductions in species richness as well as evenness in sites with Marcellus shale activity. Beta diversity analyses revealed distinct microbial community structure between sites with and without Marcellus shale activity. For example, operational taxonomic units (OTUs) within the Acetobacteracea, Methylocystaceae, Acidobacteriaceae, and *Phenylobacterium* were greater than three log-fold more abundant in MSA+ sites as compared to MSA− sites. Further, several of these OTUs were strongly negatively correlated with pH and positively correlated with the number of wellpads in a watershed. It should be noted that many of the OTUs enriched in MSA+ sites are putative acidophilic and/or methanotrophic populations. This study revealed apparent shifts in the autochthonous microbial communities and highlighted potential members that could be responding to changing stream conditions as a result of nascent industrial activity in these aquatic ecosystems.

## Introduction

Headwater streams are central to ecosystem functioning, and they are particularly sensitive to anthropogenic disturbances due to the combination of direct pollutant inputs to the watershed and the transmission of impacts from adjacent riparian terrestrial ecosystems (Sweeney, 1992; Lemke et al., 1997; Pusch et al., 1998; Lemke and Leff, 1999; Maloney and Weller, 2011; Janisch et al., 2012; Webber, 2012; Ding et al., 2013). However, the importance of headwater streams on downstream ecosystem health has only recently received attention. Low-order stream ecosystems provide habitats for unique and local communities, and impact diversity (Meyer et al., 2007) and quality of regional freshwater ecosystems (Peterson et al., 2001; Alexander et al., 2007; Freeman et al., 2007; Wipfli et al., 2007). In particular, aquatic microbial communities are central to energy flow within these ecosystems (Peterson et al., 2001; Findlay et al., 2002; Gulis and Suberkropp, 2003; Hall and Tank, 2003; Puddu et al., 2003; Wright and Covich, 2005; Hall et al., 2012; Schelker et al., 2012). The first biotic response to environmental perturbations can be seen at the lowest trophic levels, as microbial communities can readily respond to changes in their surrounding abiotic environments. Aquatic microbial community structure changes in response to biogeochemical alterations from anthropogenic sources, including agricultural, industrial, and recreational activities (Wassel and Mills, 1983; Clivot et al., 2013; Sun et al., 2013). Despite recent increases in prevalence, the impact of unconventional natural gas extraction, referred to as hydraulic fracturing or fracking, on headwater stream ecosystems has yet to be evaluated.

The mechanics and process of hydraulic fracturing and modern shale gas development has been previously described (Hubbert and Willis, 1954; Arthur et al., 2008; Ground Water Protection Council and ALL Consulting, 2009). Briefly, fracking involves drilling first vertically, then horizontally, toward a gas-bearing formation. Once the horizontal well is drilled, a combination of water, sand, and chemicals is injected at high pressure, fracturing the target formation to efficiently recover natural gas. Recent technological advances and economic conditions have favored the development of gas-bearing shale formations within the United States. When fracking occurs in the Marcellus shale formation, the resulting activity is described as Marcellus shale activity. As one of the largest shale gas formations in the United States, the natural gas extraction from the Marcellus shale has revitalized the energy industry in the northeastern United States and as a result, the state of Pennsylvania has fostered the fastest growing natural gas industry in the United States^1^ ^,^^2^ (United States Energy Information Administration, 2012). Accordingly, the rapid development of hydraulic fracturing in Pennsylvania likely will provide a variety of intriguing challenges and opportunities to investigate.

Numerous environmental surveys have postulated that hydraulic fracturing may lead to increased risks for groundwater (Davies, 2011; DiGiulion et al., 2011; Warner et al., 2012; Jackson et al., 2013; Vengosh et al., 2014), surface-water (Øvreås and Jensen, 1998; Entrekin et al., 2011; Barbot et al., 2013; Olmstead et al., 2013), and air pollution (Pacsi et al., 2013; Roy et al., 2014). Surface waters located near shale gas wells have a particularly high risk of being impacted directly or indirectly by natural gas activities (Entrekin et al., 2011; Barbot et al., 2013; Olmstead et al., 2013). Nearly 700 violations were issued by the Pennsylvania Department of Environmental Protection (PADEP) to shale gas companies from 2008 to 2010 for surface water pollution (Entrekin et al., 2011). The effects of spills, well blowouts, and storage leaks on surface water are not well known due to a lack of empirical measurement and due to the variable and unknown composition of fracking fluids (Entrekin et al., 2011; Waxman et al., 2011). Land-use alterations within the Marcellus shale region will also likely have an impact on surface water quality, especially in small headwater ecosystems. Drohan et al. (2012) identifies that core forest, defined as forest situated more than 100 meters from cleared area, is of particular risk to land use changes by Marcellus shale activity through road construction and pad development (Drohan et al., 2012). On average, 1.2–2.0 ha of land are used to construct a wellpad (3.5 ha if additional infrastructure such as roads and pipelines are considered) (Johnson et al., 2010; Grant et al., in press). Changes in stream water quality can occur from increased overland flow associated with forest disturbance (Sollins and McCorison, 1981; Meyer and Tate, 1983; Bryce et al., 2010; Jardine et al., 2012; Schelker et al., 2012; Palviainen et al., 2014). Thus, these alterations in land use within forested headwater ecosystems are likely to have major effects on stream conditions and the communities they support.

Despite potential environmental disruptions, few investigations have been published that examine the ecology of stream systems in the context of hydraulic fracturing. Further, there are no existing studies investigating the potential effects of fracking on aquatic microbial communities. While some limited metagenomic analyses of fracturing fluids and flowback waters have identified potential microbial contaminants of wells and associated infrastructure (Struchtemeyer et al., 2011; Murali Mohan et al., 2013; Wuchter et al., 2013), nothing is currently known about the impacts of fracking on surrounding environmental microbial communities. In this study, we applied microbial community analysis to headwater stream ecosystems with different histories of fracking, specifically focusing on differences between sites with no fracking activity and those with activity. We used high-throughput sequencing of the 16S rRNA gene to analyze the microbial community structure of water, sediment, bryophyte, and biofilm samples from 26 headwater stream sites in northwestern Pennsylvania. For the first time, this study revealed apparent shifts in aquatic microbial communities impacted by fracking and highlighted potential sentinel taxa that could be responding to changing watershed conditions as a result of Marcellus shale activity.

## Materials and methods

### Site selection

Stream study sites were all located on public lands, and appropriate permits were acquired through the Department of Conservation and Natural Resources (http://www.dcnr.state.pa.us) and the Pennsylvania Game Commission, SFRA-1322 (http://www.pgc.state.pa.us/portal/server.pt/community/pgc/9106). Permits were acquired through the PA Fish and Boat Commission (Permit #604) to conduct all aquatic research described. All permits are available upon request at Juniata College.

Twenty-six Pennsylvanian watersheds with unconventional shale gas well permits (PADEP, 2012) were selected for study based on the following criteria. (i) they were forested watersheds with little or no prior anthropogenic activity, (ii) they were headwater streams, (iii) they had naturally-reproducing trout species [*Salvelinas fontinalis* and *Salmo trutta*], and (iv) they were located within the Marcellus shale region in northwestern Pennsylvania. Figure S1 shows stream sampling locations. Streams without fracking infrastructure development prior to sampling were grouped as lacking Marcellus shale activity (MSA−, *n* = 10). Stream sites with at least one wellpad were considered to exhibit Marcellus shale activity (MSA+, *n* = 16), except in the cases of Trout Run and Deer Run. An Unnamed Tributary to Trout Run and Alex Branch are tributaries of Trout Run that exhibit Marcellus shale activity. Because these two MSA+ tributaries feed into Trout Run, it is included in the MSA+ group. Deer Run did not have any drilled wells in its watershed, but wellpad construction began prior to sampling. Therefore, Deer Run was classified as exhibiting Marcellus shale activity. Table 1 provides information regarding the extent of Marcellus shale activity within the sampled watershed. It should be noted that two streams (Little Laurel Run and Alex Branch) had documented hydrofracking-related contamination occur within their watershed prior to our sampling efforts according to the Pennsylvania Department of Environmental Protection (PADEP, 2012). Detailed information about the sample sites and watershed characteristics are further described in Grant et al. and have been summarized in the Supplementary Information (Grant et al., in press). The similarity of the stream and watershed characteristics of the selected streams make these sites ideal to compare with respect to the impacts of Marcellus shale activity (Grant et al., in press).

**Table 1:** **Watershed information about streams sampled in this study**.

**Stream**	Impact status^*^	**Number of wells**	**Number of wellpads**	**Distance to nearest well (meters)**	**River basin**
Alex branch	MSA+	10	9	1010	West Branch of the Susquehanna
Bear run	MSA+	2	1	1193	Clarion River
Ben's creek	MSA−	0	0	0	Stonycreek River
Big break hollow	MSA−	0	0	0	Juniata River
Camp run	MSA−	0	0	0	Allegheny River
Coldstream run	MSA+	12	5	970	West Branch of the Susquehanna
Crooked run	MSA−	0	0	0	West Branch of the Susquehanna
Dead man's lick	MSA−	0	0	0	West Branch of the Susquehanna
Deer run	MSA+	0	2	0	West Branch of the Susquehanna
Diamond run	MSA−	0	0	0	Juniata River
Dixon run	MSA+	0	2	0	West Branch of the Susquehanna
East beaver run	MSA+	0	0	0	Allegheny River
Findley run	MSA−	0	0	0	Conemaugh River
Indian run	MSA+	12	2	1738	Allegheny River
Iron run	MSA+	2	1	3398	Allegheny River
Laurel run	MSA+	2	1	3188	Clarion River
Lick run	MSA+	10	3	1470	West Branch of the Susquehanna
Little laurel run	MSA+	26	11	1887	West Branch of the Susquehanna
Little wolf run	MSA+	4	2	2481	Clarion River
Long run	MSA+	4	2	2063	Clarion River
South Branch North Fork Redbank Creek	MSA+	1	1	1065	Allegheny River
Stone run	MSA+	19	5	538	West Branch of the Susquehanna
Straight creek	MSA−	0	0	0	Clarion River
Trout run	MSA+	12	2	1430	West Branch of the Susquehanna
Un-named Tributary to the Clarion River	MSA−	0	0	0	Clarion River
Vineyard run	MSA−	0	0	0	Clarion River

* MSA+ denotes presence of Marcellus shale activity, while MSA− represents streams with no Marcellus shale activity in the watershed.

### Field sampling

Samples (*n* = 58) were collected from bryophyte, sediment, biofilm, and water matrices. Aquatic mosses (*n* = 24) were cut directly from submerged rock substrates with a sterile scalpel to collect their microbial communities. Moss samples, consisted of two common water mosses, *Fontinalis sphagnifolia* and *Fontinalis antipyretica*. Sediment samples (*n* = 24) located adjacent to the water-bank interface were collected using sterile scoops. Biofilm samples from South Branch North Fork Redbank Creek and Little Laurel Run (*n* = 2) were collected in sterile 50 mL conical tubes. For water samples (*n* = 8), 1 liter of stream water was collected from a central riffle using a sterile Nalgene bottle. Water samples were filtered on site with 0.22 μm polyethersulfone filters (Millipore, Billerica, MA) and stored in sterile Whirl-Pak bags (Nasco, Fort Atkinson, WI). All samples were immediately placed on dry ice then stored at −80°C. Stream water chemistry measurements [pH, conductivity, salinity, total dissolved solids (TDS), and temperature] were taken on site at the time of sampling with a PCSTestr 35 (Oakton Instruments, Vernon Hills, IL) that was calibrated weekly. For later analysis of organic matter content (DOC) and nitrogen concentration, water samples were collected in pre–cleaned amber glass bottles and pre–cleaned 500 ml polyethylene (HDPE) bottles and stored on ice. Water samples were collected upstream of microbial water samples and at the centroid of flow in riffles under baseflow conditions.

### DNA extraction

Prior to nucleic acid extraction, 0.4 g of bryophyte material from each site was transferred to sterile centrifuge tubes and 4 mL of phosphate buffered saline solution (1X PBS) was added. The samples were vortexed for 15 s and centrifuged at 4000 × g at 4°C for 5 min. The supernatants were centrifuged at 16,000 × g for 10 min at 4°C and the resulting cell pellets were resuspended in 200 μL of 1X PBS. Nucleic acid extractions were performed on bryophyte-derived cell pellets, 0.6 g of sediment and biofilm samples, and water filters using a modified Cetyltrimethyl ammonium bromide (CTAB) Phenol/Chloroform/Isoamyl-alcohol method as described in (Hazen et al., 2010). A more detailed description of the extraction procedure can be found in the Supplementary Information.

### Illumina tag PCR

Duplicate 25 μL Illumina tag Polymerase Chain Reactions (PCR) from each sample (*n* = 58) contained final concentrations of 1X PCR buffer, 0.8 mM dNTP's, 4 μ M 515F Illumina barcoded forward primers, 4 μ M 806R reverse primers, 0.25 U Taq Polymerase per reaction, and 10 ng of template DNA per reaction. PCR was carried out on a MJ Research PTC-200 thermocycler (Bio-Rad, Hercules, CA) using the following cycling conditions: 98°C for 3 min; 25 cycles of 98°C for 1 min, 55°C for 40 s, and 72°C for 1 min; 72°C for 10 min; and kept at 4°C. PCR products were visualized on a 2% E-gel (Life Technologies, Carlsbad, CA). Library purification, verification, and sequencing of libraries are described in the Supplementary Information.

### Bioinformatics and statistical analyses

Sequence data for this project can be found in NCBI's Short Read Archive under accession number SRP046758. Due to a quality score drop at 98 bp on reverse reads, only the forward reads were analyzed. Sequences were trimmed at a length of 120 bp and quality filtered at an expected error of less than 1% using USEARCH v7 (Edgar, 2013). After quality filtering, reads were analyzed using the QIIME 1.7.0 software package (Caporaso et al., 2010; Caporaso and Lauber, 2011). Chimeric sequences were identified using USEARCH61 (Edgar, 2010). A total of 3.2 million sequences were retrieved after quality filtering and chimera checking. Open reference operational taxonomic units (OTUs) were picked using the USEARCH61 algorithm (Edgar, 2010), and taxonomy assignment was performed using the Greengenes 16S rRNA gene database (13-5 release, 97%) (DeSantis et al., 2006). Sequences that did not match the database were subsequently clustered using de novo clustering. A detailed description of alpha and beta diversity analyses can be found in the Supplementary Information. Visualization of trends in microbial community structure for MSA+ and MSA− samples were generated in R using the *Phyloseq* (McMurdie and Holmes, 2013; R Core Team, 2014) and details are described in the Supplementary Information.

LEfSe was used to identify taxonomic biomarkers between MSA− and MSA+ communities (Segata et al., 2011) and for intra-matrix comparisons. Genus-level relative abundances were multiplied by 1 million and formatted as described in Segata et al. (2011). Comparisons were made with “Impact Status” (MSA+ or MSA−) as the main categorical variable (“Class”) and “Sample Matrix” (sediment, bryophyte-associated, or water) as the secondary categorical variable (“Subclass”). Alpha levels of 0.05 were used for both the Kruskal–Wallis and pairwise Wilcoxon tests. Linear Discriminant Analysis (LDA) scores for the top 10 features from each class were plotted.

Statistical analysis of stream water pH, conductivity, TDS, salinity, and temperature was conducted between MSA+ and MSA− streams using *t*-test and Kruskal–Wallis tests in Minitab (v.16). Data was transformed (log_10_) to help meet the assumptions of ANOVA, while a non-parametric Kruskal–Wallis test was used for stream pH comparisons. All statistical analyses were considered significant at α = 0.05. Pairwise comparisons of bacterial community structure between counties, based on bray-curtis and unweighted UniFrac distance metrics, were generated using Phyloseq on an unrarified OTU table. All samples within the object were merged based on county, after which the “distance” function was used to generate all pairwise comparisons.

Spearman correlations were calculated to examine the relationship between continuous abiotic variables and microbial community composition at the genus-level. Due to inherent correlations among abiotic factors, an appropriate p-value correction was not apparent. Instead, the top 10 most positive and negative correlations between genera and pH and number of wellpads were selected for visualization.

## Results

### Watershed and stream measurements

A comparison of water chemistry revealed that pH was the only significantly different parameter between MSA+ and MSA− streams (median pH: MSA+ = 6.9; MSA− = 7.7; Wilcoxon rank sum test, *p* = 0.007). Two of the 26 sample sites, Little Laurel Run (pH = 4.55) and Alex Branch (pH = 4.88), had documented spills of fracking fluid and had the lowest pH of all watersheds characterized in this study. All other collected measures (conductivity, TDS, salinity, temperature, DOC, total nitrogen, and elevation) did not show a significant difference between MSA+ and MSA− streams (Wilcoxon rank sum test; *p* > 0.05 for all comparisons). Additionally, pH was negatively correlated to the number of wellpads present within a watershed (Spearman's rho = −0.72, *p* ≤ 0.0001), but no other measures strongly correlated with number of wellpads (absolute value of Spearmans's rho = 0.43, *p* > 0.05 for all other comparisons). The number of wellpads in MSA+ watersheds ranged from 0–11, and there was a maximum of 26 wells in a given watershed (Table 1). Comparison of watershed characteristics showed that watershed land cover (% agriculture, % forested, % wetlands, and forest composition) was not significantly different between MSA+ and MSA− watersheds (Grant et al., in press).

### Microbial community results

**Figure 1:**
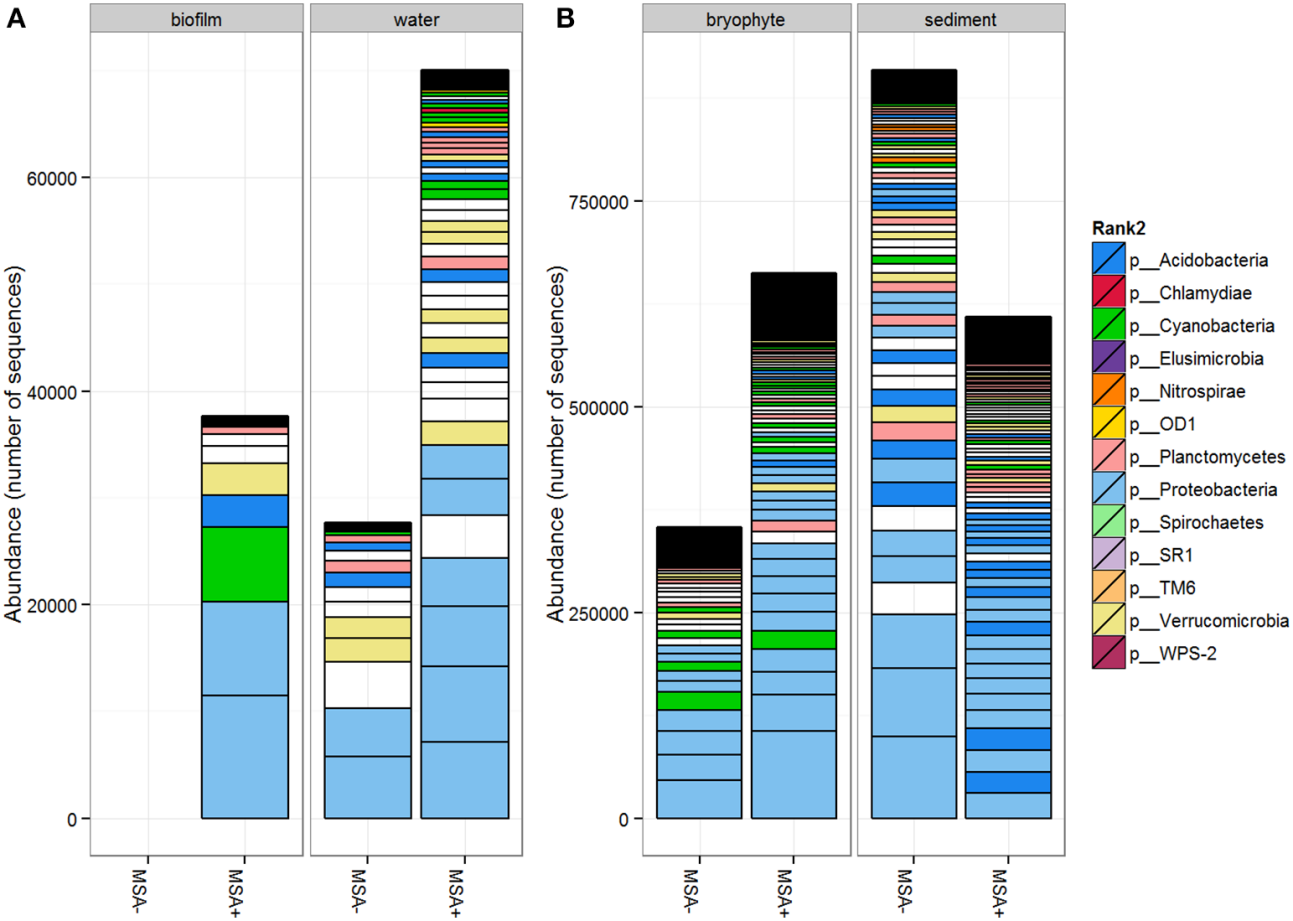
**Abundance of bacterial phyla in (A) biofilm (*n* = 2) and water (*n* = 8) samples, (B) bryophyte (*n* = 24), and sediment (*n* = 24) samples**. Samples were grouped by Marcellus shale activity status and by sample matrix. OTUs with more than 20 sequences in at least one sample are shown. Plots were created in phyloseq (McMurdie and Holmes, 2013) using the functions tax_glom and tip_glom (height = 0.9) to consolidate the taxa. Unknown phyla are shown in white. All biofilm samples were collected from MSA+ sites. Note that the difference in sequence abundance is attributed to number of samples and sequencing depth.

Phylum-level community structure for MSA+ and MSA− samples within each sample matrix showed that Proteobacteria was the dominant phylum across all samples (Figure 1). No major shifts in phyla abundance were noted between MSA+ and MSA− sites for sediment, water, and bryophyte samples (Figures S2A–S2C). However, major changes in community structure at the phylum rank were observed in the two biofilm samples from Little Laurel Run (*n* = 13 wellpads) and South Branch North Fork Redbank Creek (*n* = 1 wellpad). The biofilm sample from Little Laurel Run, a spill site, was dominated by multiple phyla, including Proteobacteria, Cyanobacteria, Verrucomicrobia, Acidobacteria, and Bacteroidetes, while the biofilm sample from South Branch North Fork Redbank Creek was exclusively dominated by Proteobacteria (Figure S2D).

Alpha diversity rarefaction curves suggested a reasonable coverage of diversity was reached, as curves approached a horizontal asymptote as sequencing depth increased (Figure S3). Based on both observed species and Chao1 alpha diversity metrics, water samples possessed the greatest species richness, followed by sediment, bryophyte, and biofilm samples respectively. Although sequencing depth of water was an order of magnitude lower than for bryophyte and sediment samples, alpha rarefaction plots showed water samples were more diverse at an even sampling depth (Figure S3).

**Figure 2:**
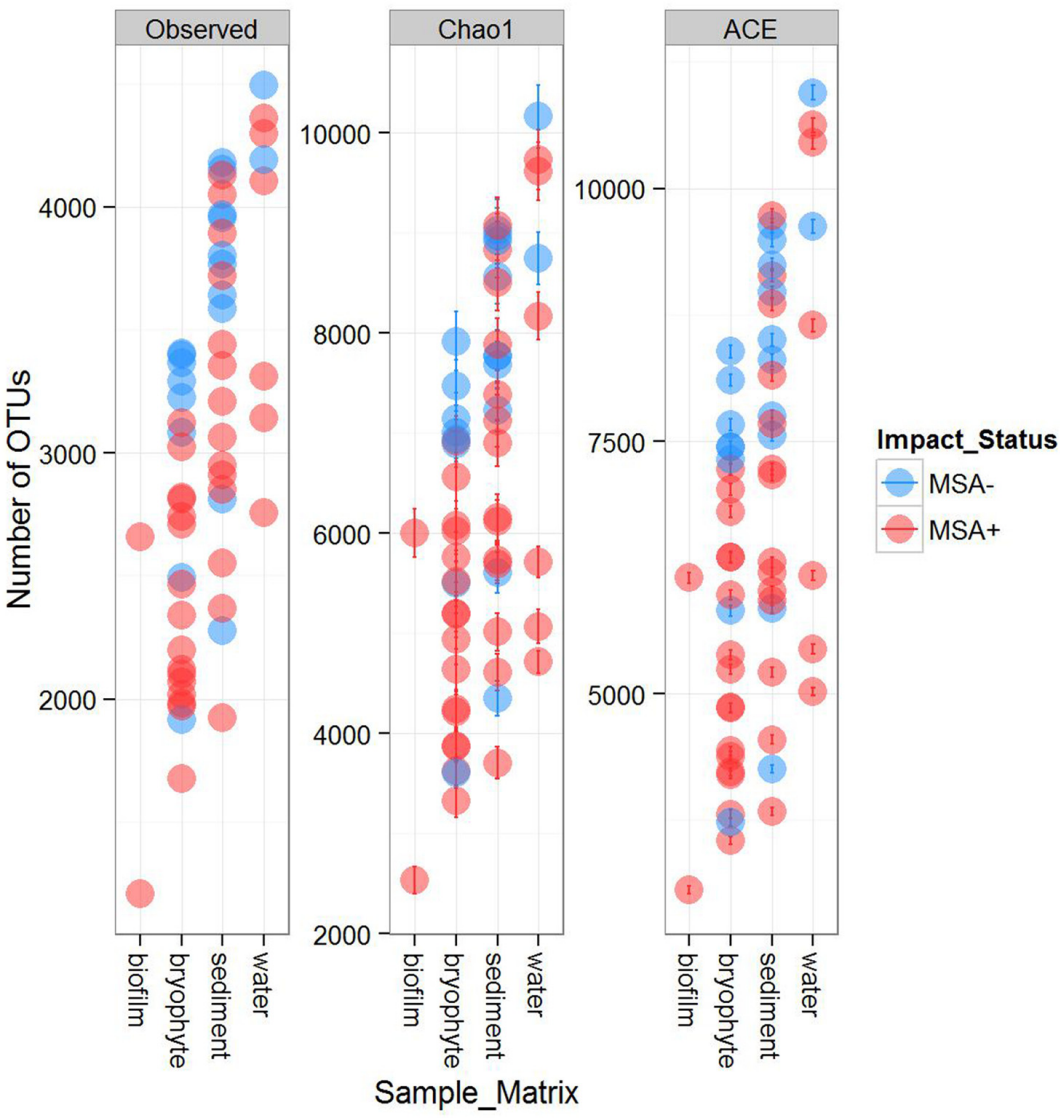
**Alpha Diversity metrics in biofilm, bryophyte, sediment, and water samples from MSA+ and MSA− sites**. Species richness was estimated by performing multiple rarefactions up to a depth of 11,200 sequences per sample. The richness of a single OTU table from the maximum rarefaction depth was estimated using observed richness, Chao1, and ACE and visualized using Phyloseq. For each metric, species richness is separated by impact status and sample matrix. Samples from impacted environments (labeled MSA+) tend to be less diverse than those from unimpacted ones.

Richness was significantly lower in MSA+ samples as compared to MSA− samples (Table 2). The number of observed species for MSA+ sites was 3450 ± 679 OTUs, while MSA− sites had an observed richness of 2858 ± 771 OTUs. Reduced richness in MSA+ samples was statistically significant down to class and phylum taxonomic ranks. Bacterial evenness also appeared to be impacted by Marcellus shale activity, as Heip's evenness measurements were significantly lower in MSA+ samples (Table 2). When comparing alpha diversity within sediment, bryophyte, and water matrices, samples from MSA+ had lower alpha diversities than MSA− samples from that same matrix (Figure 2). Bryophyte-associated microbial communities from MSA+ samples had significantly lower alpha diversity than MSA− samples (Non-parametric two-sample *t*-test, *p* = 0.021), while water and sediment alpha diversity comparisons were not statistically different.

**Table 2:** **Alpha diversity comparisons of MSA+ and MSA− communities across taxonomic ranks**.

**Metric**	**Species**	**Genus**	**Family**	**Order**	**Class**	**Phylum**
Observed OTUs	0.003^*^	0.005^*^	0.001^*^	0.005^*^	0.009^*^	0.027^*^
Chao1	0.002^*^	0.003^*^	0.006^*^	0.003^*^	0.027^*^	0.088^*^
Heip's evenness	0.015^*^	0.054	0.12	0.169	0.253	0.743

* Indicates significant p-values non-parametric two sample t-test with 999 Monte Carlo permutations, α = 0.05.

Bacterial diversity also shared significant relationships with environmental parameters. For example, the number of wellpads in each watershed had a strong negative correlation to alpha diversity (Spearman's rho = −0.551, *p* < 0.00001) while pH had a strong positive correlation with alpha diversity (Spearman's rho = 0.592, *p* < 0.00001). It should be noted that the number of wellpads present and pH also were negatively correlated to each other (Spearman's rho = −0.72, *p* < 0.0001). Bacterial communities from sites with documented fracking fluid releases (Little Laurel Run and Alex Branch) had much lower alpha diversity as compared to average alpha diversity metrics calculated for MSA+ sites without spills and MSA− watersheds (Table 3). For example, the sediment and water samples from MSA− watersheds had nearly two times more observed species as compared to the two spill sites (Table 3). As mentioned above, these two spill sites also had the lowest pH of all streams evaluated in this study.

**Table 3:** **Alpha diversity measures for biofilm sediment and water samples collected from streams with fracking spills**.

**Sample matrix**	**Watershed**	**Group**	**Heip's evenness**	**Observed Species**	**Chao1**
Biofilm	Little Laurel Run	Spill	0.0691	2587	5122.05
	SBNFRC	MSA+	0.0079	1210	2657.26
Sediment	Alex Branch	Spill	0.1092	2378	4645.48
	Average (±SD)	MSA+	0.1437 ± 0.0172	3157.67 ± 342.11	6520.81 ± 847.95
	Average (±SD)	MSA−	0.1622 ± 0.0172	3660.91 ± 338.87	7659.47 ± 899.97
Water	Little Laurel Run	Spill	0.1204	2762	5045.77
	Average (±SD)	MSA+	0.1629 ± 0.0246	3746 ± 557.16	7112.29 ± 2081.44
	Average (±SD)	MSA−	0.1698 ± 0.0414	4377 ± 214.14	9578.97 ± 759.96

**Figure 3:**
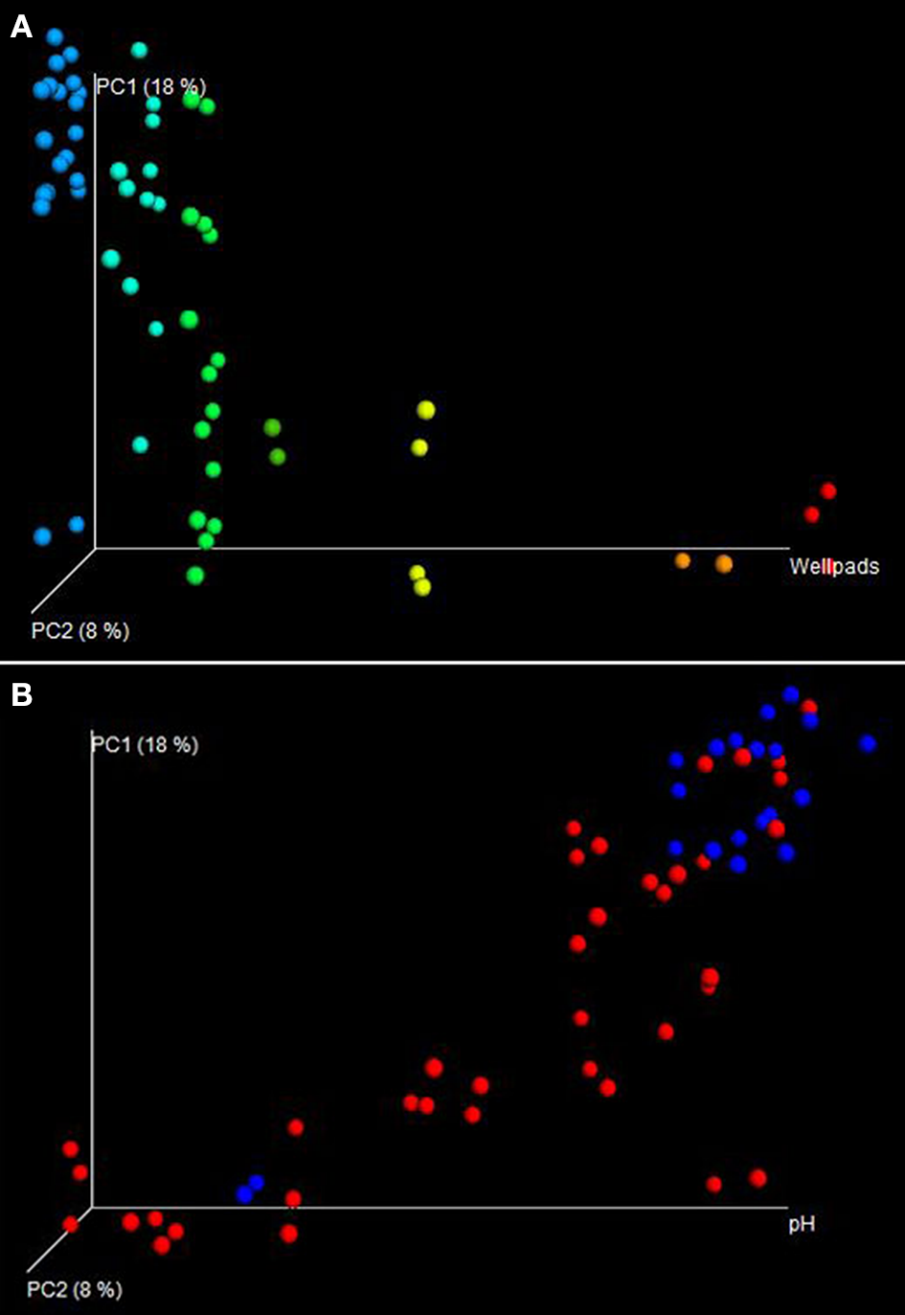
**Directional Principal Coordinates Analysis (PCoA) plots were used to visualize differences in weighted UniFrac distances of MSA+ and MSA− samples. (A)** Samples were plotted according to number of wellpads along the horizontal axis of the directional PCoA plot. Samples with no wellpads are colored in blue, whereas samples with the highest wellpad count are colored red. Distinct clustering can be observed between samples with a high number of wellpads and samples with a low number of wellpads. A majority of samples with a low wellpad count cluster at the top of the PC1 axis, a region where no samples with a high wellpad count are observed. **(B)** When imposing pH to the horizontal axis, distinct clustering between MSA+ (red) and MSA− (blue) is observed, implying pH in conjunction with impact status shapes microbial community structure.

Beta diversity analyses of microbial communities revealed distinct microbial community structure between MSA+ and MSA− samples (Figure 3). A directional PCoA plot generated using weighted Unifrac distances showed distinct clustering of samples based on number of wellpads (Figure 3A) (Vázquez-Baeza et al., 2013). Samples with a high number of wellpads clustered together. Samples with less wellpads cluster higher on the principal coordinates axis 1, a region where no samples with a high wellpad count are observed. A directional PCoA plot with a strong pH gradient displayed distinct clustering of MSA+ and MSA− samples, suggesting that bacterial community structure is shaped by both pH and fracking status of that watershed (Figure 3B). Because sample matrix explained the most (38%) variation in beta diversity across all samples (adonis; Pr > *F* = 0.001), within-matrix beta diversity statistics were performed. When analyzing each sample matrix separately, sediment (ANOSIM; *p* = 0.038) and bryophyte (ANOSIM; *p* = 0.016) matrices showed distinct clustering of MSA+ and MSA− samples (Figure S4). The number of wellpads accounted for a significant portion of variation in all sample matrices. Within sediment, bryophyte, and water samples the number of wellpads in a watershed explained 20.5% (Adonis; Pr > *F* = 0.002), 14.2% (Adonis; Pr > *F* = 0.001), and 20.3% (Adonis; Pr > *F* = 0.024) of variation in beta diversity, respectively. pH also explained a large amount of variation in beta diversity, as it accounted for 25.8% (Adonis; Pr > *F* = 0.001), 20.4% (Adonis; Pr > *F* = 0.001), and 28.8% (Adonis; Pr > *F* = 0.001) in sediment, bryophyte, and water matrices, respectively.

**Figure 4:**
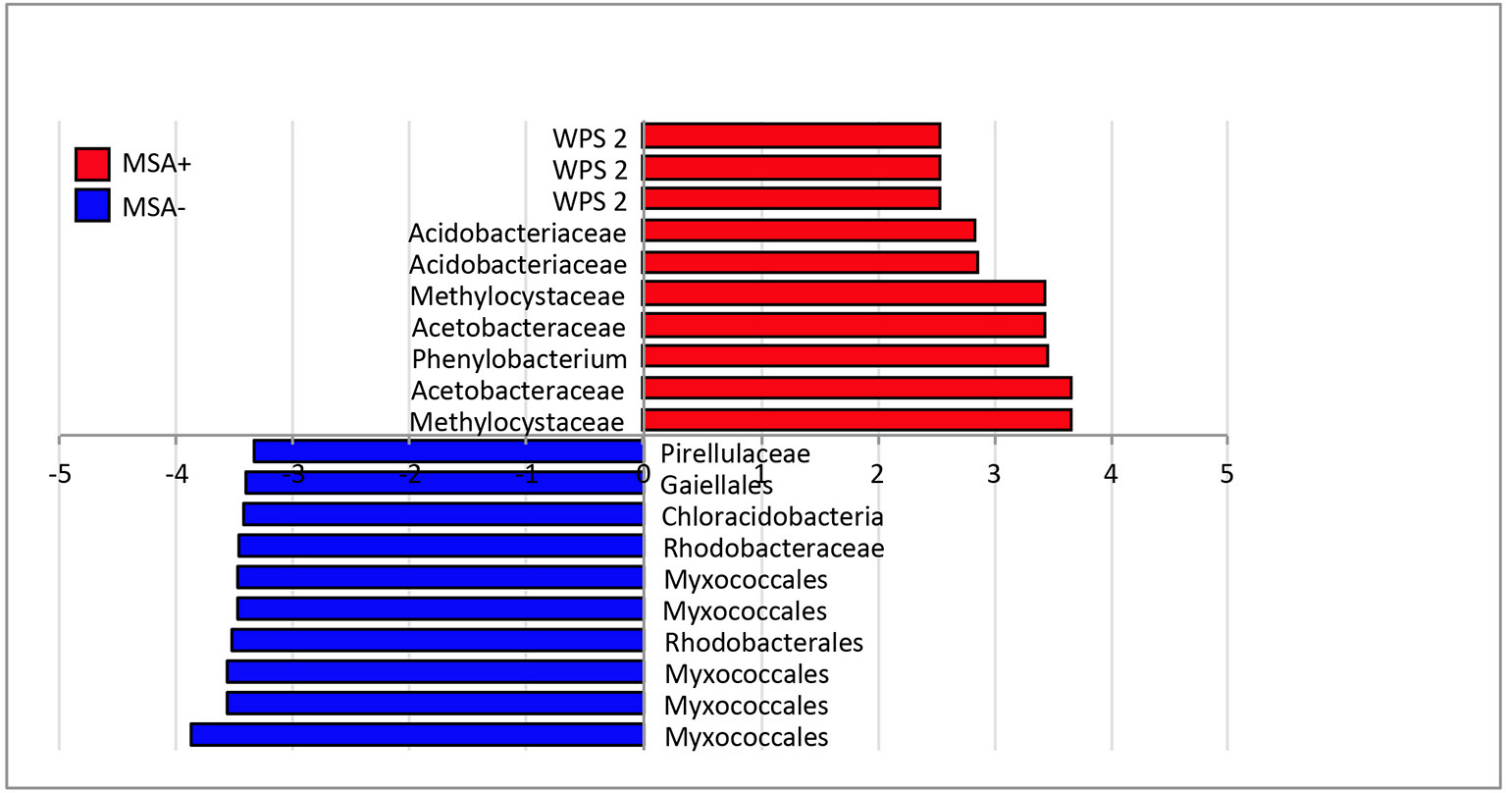
**LEfSe plot of taxonomic “biomarkers” of MSA+ and MSA− communities**. Here, the top 10 most differentially significant taxa of each group (MSA+/MSA−) are plotted, where red bars represent taxa significantly enriched in MSA+ sites and blue bars signify taxa more abundant in MSA− streams. Features plotted on a logarithmic scale according to the experimental group to which they were significantly associated. LEfSe utilizes Kruskal–Wallis tests to determine significantly different taxonomic features (α ≤ 0.05) between experimental groups, a pairwise Wilcoxon rank sum statistic to test biological consistency across subgroups (α ≤ 0.05), and finally a linear discriminant analysis to determine the effect size, or magnitude of variation of the features between groups. Features are plotted on a logarithmic scale according to the experimental group to which they are significantly associated.

While differences in microbial community structure were not seen between MSA+ and MSA− sites at the phylum rank, significant changes were observed at a finer phylogenic resolution. For example, biomarker analyses revealed that Methylocystacea, Acetobacteraceae, *Phenylobacterium*, Acidobacteriaceae and WPS-2 groups were amongst the most significantly enriched taxa in MSA+ samples (Figure 4). Biomarker analysis performed on individual matrices also supported similar trends (Figures S5–S7). Methylocystaceae, Acetobacteraceae, *Phenylobacterium* were > 3 log_10_-fold more abundant, and WPS-2 and Acidobacteriaceae were > 2 log_10_-fold more abundant in MSA+ streams. Five unknown taxa within the order Myxococcales were >3 log_10_-fold higher in abundance in MSA− streams.

**Figure 5:**
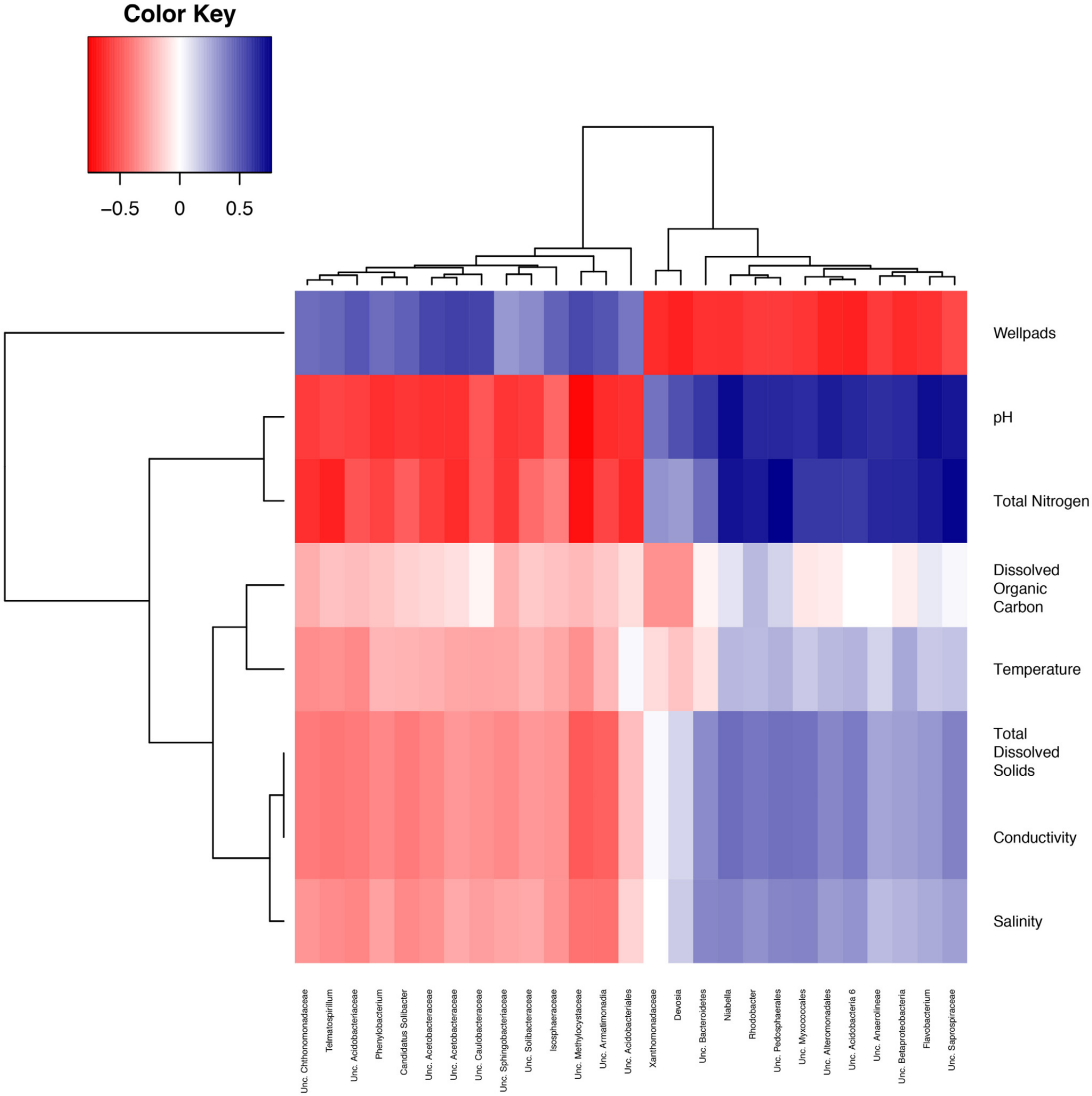
**Spearman correlation of abiotic factors with bacterial genera**. Spearman correlations were calculated between each genus and several abiotic factors. This heatmap displays the top 10 genera most positively and most negatively correlated with each pH and number of wellpads, respectively. Each cell represents the Spearman rho value for the correlation between a genus (columns) and abiotic factor (rows) ranging from red (for negative correlations) to blue (for positive correlations) with small absolute rho values represented by white. Hierarchical clustering was used to place each genera and abiotic factors with similar relationships near each other.

Many of these enriched bacterial taxa also shared strong relationships with both pH and the number of wellpads in a watershed. Spearman correlations revealed a negative relationship between the Acidobacteriaceae and pH and a positive relationship of this taxa with the number of wellpads (Figure 5). In all cases, OTUs strongly correlating with wellpads correlated negatively to pH and total nitrogen concentrations (pH absolute Spearman's rho = 0.75–0.58; wellpad absolute Spearman's rho = 0.44–0.57), including unclassified Methylocystaceae, Armatimonadia, Sphingobacteriaceae, Candidatus *Solibacter*, Chthonomonadaceae, Solibacteraceae, Caulobacteraceae, Acidobacteriaceae, Isosphaeraceae, Soilbacteraceae, two genera within the Acetobacteraceae, *Phenylobacterium*, and *Telmatospirillum* (Table S1). Furthermore, OTUs correlating negatively with wellpads correlated positively to pH and total nitrogen concentration (pH absolute Spearman's rho = 0.63–0.73; wellpad absolute Spearman's rho = 0.66–0.59) including unclassified Saprospiraceae, Alteromonadales, Pedosphaerales, Betaproteobacteria, Myxococcales, Anaerolineae, Xanthomonadaceae, Bacteroidetes, and the genera *Devosia*, *Rhodobacter*, *Niabella*, and *Flavobacterium*.

Comparing the impact of spatial variation on the microbial community structure showed that there was not much clustering of the samples by county with the exception of Clearfield and Elk counties (Figure S8). Clearfield county, which contains both spill sites, and nine MSA+ sites, also feeds into streams in Elk county (Figure S1). Both of these counties possessed similar microbial community structures, and were different compared to all other counties based on both bray-curtis and unweighted unifrac metrics (Table S2). In addition, samples collected from both Elk and Clearfield counties were most dissimilar from all other counties sampled in this study.

## Discussion

Twenty-six headwater stream ecosystems in Pennsylvania were studied to evaluate the potential impacts of unconventional natural gas extraction on aquatic microbial communities. High-throughput sequencing data enabled deep coverage of the diverse microbial communities in these aquatic environments, which facilitated a detailed analysis of the variation in bacterial diversity and community structure along environmental gradients. As detailed below, microbial community analyses revealed marked differences in bacterial diversity in watersheds with and without fracking. Additionally, the relative abundance of certain bacterial taxa correlated with pH gradients and the amount of fracking development in a given watershed.

The stream water chemistry data revealed that streams located in watersheds with Marcellus shale activity had significantly lower pH than sites with no activity. Observed differences in stream pH can be attributed to variation in watershed characteristics, disparities in acid rain deposition, or fracking activities (Grant et al., in press). A thorough GIS survey revealed no significant difference in watershed characteristics in the sites evaluated in this study (Grant et al., in press). Increased acidification by atmospheric deposition would have resulted in concomitant increases in stream water nitrogen concentration, which was not significantly different between MSA+ and MSA− watersheds (Baker et al., 1996; Grant et al., in press). The lower observed pH in watersheds with Marcellus shale activity might be attributed to exposure of pyritic geological formations by the drilling process, as weathering of acid rock has attributed to stream acidification in other scenarios (Hammarstrom et al., 2005; Fierer and Jackson, 2006). Further, a number of concentrated acids (and bases) can be used in the hydraulic fracturing process, and fracking fluid mixtures can themselves be highly acidic, though many of these fluid formulations still remain unknown or undisclosed by industry. The negative correlation between number of wellpads within a watershed and pH suggests that fracking may be directly or indirectly increasing the acidity of headwater stream ecosystems. Further, the fact that no other measured stream characteristics, other than pH, differed between MSA+ and MSA− sites, suggests that our analyses are accurate reflections of the effects of activity, not artifacts related to differences in the sites selected for this study.

Alpha diversity analyses indicated lower diversity in aquatic bacterial communities in streams with Marcellus shale activity as compared to streams with no activity (Figure 2 and Table 2). When comparing alpha diversity within each sample matrix, bryophyte samples had the most significant difference between MSA+ and MSA− sites. Reduction in bacterial richness associated with these bryophyte samples could be related the sensitivity of moss to environmental perturbations, as several moss species are common bio-indicators of environmental quality. It is possible that fracking is indirectly affecting alpha diversity by directly affecting pH. Although not well studied in headwater streams, this finding is supported by previous research, which showed acidic pH was associated with lower bacterial diversity in soils (Baker et al., 1996; Hammarstrom et al., 2005). While this is the first study to assess the impacts of fracking on bacterial communities in aquatic environments, numerous other studies have demonstrated the detrimental impacts a variety of anthropogenic activities have on bacterial diversity in the environment (Wassel and Mills, 1983; Clivot et al., 2013; Sun et al., 2013). However, it should be noted that other environmental factors not measured in this study could also be contributing to decreased observed alpha diversities.

Significant differences in beta diversity were also observed between MSA+ and MSA− sites, suggesting fracking maybe impacting microbial community structure in these aquatic environments. When comparing beta diversity within each sample matrix, bryophyte samples observed the greatest difference in phylogenetic distance between MSA+ and MSA− samples. (Figure S4). This, in congruence with the distinct differences in alpha diversity for bryophyte samples, further suggests that the microbial community associated with bryophyte may be sensitive to potential perturbations in these environments. As aquatic microbial communities are central to ecosystem functioning in headwater streams (Peterson et al., 2001; Findlay et al., 2002; Gulis and Suberkropp, 2003; Hall and Tank, 2003; Wright and Covich, 2005; Hall et al., 2012; Schelker et al., 2012), it is imperative to track the potential response of these aquatic microbial communities to Marcellus shale activities. The number of wellpads in a watershed shared a strong relationship with beta diversity, suggesting increasing development could be shaping microbial community structure in headwater streams via increased land alteration or potential fracking fluid releases. Detailed investigation revealed specific taxa, including the Methylocystaceae, Acetobacteraceae, WPS-2, and *Phenylobacterium* were enriched in MSA+ sites (Figure 4). Interestingly, several of these taxa also shared strong correlations with pH and number of wellpads in a watershed, suggesting the relationship of these taxa to environmental changes (Figure 5). Increased acidity is known to impact aquatic ecosystem structure at microbial (Mulholland et al., 1992; Dangles and Gessner, 2004; Rousk et al., 2010) and higher trophic levels (Mulholland et al., 1992; Fierer et al., 2007; Simon et al., 2009; Rousk et al., 2010) and may be the mechanism responsible for the increased abundance of potential acidophilic taxa identified in MSA+ sites, such as the Acidobacteriaceae, Acetobacteraceae, and Methylocystaceae. This is particularly clear when considering that the two of the most acidic streams (contaminated by spills) have very high abundance of acidobacterial OTUs, including the Koribacteraceae (11%) and acid-tolerant iron-oxidizers of the genus *Gallionella* (6%).

Interestingly, many of the taxa enriched in MSA+ sites (i.e., OTUs within the Methylocystaceae and Acetobacteraceae) also have methanotrophic capabilities (McKnight and Feder, 1984; Liebner et al., 2009). Recent studies have reported increased groundwater methane concentrations with proximity to natural gas drilling and hydraulic fracturing sites (Davies, 2011; Jackson et al., 2013). Streams in these watersheds are primarily first order streams and are thus fed mostly by groundwater. Increases in abundance of methanotrophic bacteria may be an early indication of increases in methane contamination. It should also be noted that there were increases in the abundance of WPS-2 taxa in MSA+ samples, and this group has been shown to co-reside with methanotrophs, suggesting WPS-2 populations might be utilizing the derivatives of methane oxidation (Uhlig et al., 1986; Nogales and Moore, 2001; Fuss and Smock, 2003; Sharp et al., 2012; Grasby et al., 2013). While high-throughput 16S rRNA gene sequencing provided insight into the potential impacts of fracking on microbial communities in the headwater stream ecosystems, future studies should address the functional capacity and metabolic response of these microbial communities to environmental perturbations associated with fracking. Future studies should focus on measuring methane concentration and using stable isotope probing to determine if the source of this methane is from natural gas stores. In addition, functional meta-omics studies will help describe the functional response of microbial communities to methane in these environments. Metagenomic, metatranscriptome, and metabolomic approaches could provide high-resolution information about the functional capacity, expression and metabolic capabilities of populations enriched and inhibited by fracking activity.

*Phenylobacterium* was found to be significantly enriched in MSA+ sites and also was positively correlated with the number of wellpads and negatively correlated with pH. Optimal growth of some species of *Phenylobacterium* has occurred on artificial compounds such as chloridazon, antipyrin, and pyramidon, though it is a common inhabitant of soil communities (Eberspacher and Lingens, 2006; Oh and Roh, 2012). Several members of this genus also grow optimally in slightly acidic conditions between pH 6 and 6.5 (Yang et al., 2014). *Phenylobacterium* may be capable of degrading phenyl-compounds and other complex hydrocarbons in acidic environments and are succeeded by other groups of hydrocarbon degraders (Oh and Roh, 2012; Marušincová et al., 2013). Interestingly, this genus was strongly positively correlated with the number of wellpads in a watershed and negatively correlated to pH. Altogether, shifts in several aforementioned taxa suggest that these populations may be responding to environmental perturbations introduced by fracking development either through land disturbances introduced by infrastructure development or potential releases of fracking fluids into the environment. However, future time-series studies will need to carefully track the potential successional changes in microbial communities in response to documented spills.

Several bacterial taxa had significantly lower relative abundance in MSA+ samples, including several OTUs within the Rhodobacteraceae, Myxococcaceae, Hyphomicrobaceae, and Xanthomonadaceae. These taxa shared very strong positive correlations with pH and strong negative correlations with the number of wellpads in a watershed, indicating these taxa may be inhibited by perturbations introduced by fracking development in these watersheds. Interestingly, these taxa are known to have denitrifying capabilities, and lower pH can result in reduction of denitrification rates (Baeseman et al., 2006). Thus, the more acidic pH associated with MSA+ sites could be one possible mechanism for the lower abundance of denitrifying populations observed in MSA− sites.

Beta diversity analyses revealed clear clustering of samples by matrix, which indicates the shifts in community structure are environment-specific. This finding is not surprising, as each microenvironment likely harbors unique conditions in which different populations thrive. When evaluating beta diversity within each matrix, samples were differentiated by Marcellus shale activity, further suggesting fracking activities could be shaping the structure within different aquatic microbial communities. Members exclusively from the Acidobacteria were enriched in sediment samples collected from MSA+ sites, while Alphaproteobacteria were enriched in bryophyte- and water-associated MSA+ samples (Figures S5–S7). As previously mentioned, several enriched taxa within these classes, share negative correlations with pH and have been previously shown to be acidophilic or acid-adapted taxa. Four members of the phylum OD1 were significantly more abundant in MSA+ water samples and the phylum OD1 has been associated with complex hydrocarbon degradation and biofilm formation (Kantor et al., 2013).

While several interesting differences were noted in diversity and bacterial community structure in sites with and without fracking activity, future studies need to address both temporal and spatial variation. An analysis of spatial variation impacts on microbial community structure showed little county-specific clustering of samples, except for streams in the two counties (Clearfield and Elk County), which encompassed streams impacted directly by, or connected to streams that experienced documented fracking fluid spills (Figure S8). It should also be noted that Clearfield County, which contained the highest number of MSA+ sites (*n* = 9), was most dissimilar to all other counties sampled in this study, further illustrating the potential impacts of fracking on microbial community structure (Table S2). While no significant differences were noted in watershed characteristics between MSA+ and MSA− sites, future studies will assess pre- and post-fracking impacts within the same stream to control for any unmeasured differences in watershed characteristics.

## Conclusions

This study represents the first investigation of the potential impact of Marcellus shale activity on aquatic bacterial communities in headwater stream ecosystems in northwestern Pennsylvania. The results of this study showed (i) reduction in the diversity of bacterial communities in streams with fracking activity and (ii) specific shifts in bacterial community structure were indicative of watershed status and correlated with changes in pH. These findings are relevant and timely, as pristine headwater stream ecosystems may bear the largest likelihood for environmental impacts and ecosystem disruption, chiefly because of the potential for relatively large land use alterations introduced in these watersheds as a result of wellpad development. While additional investigation and long-term studies will be necessary to fully elucidate the impacts of Marcellus shale activity on aquatic ecosystems, this study serves to provide baseline bacterial community data for future studies. Future work should focus on additional chemical measurements including isotopic carbon measures, and meta-omics analyses, which will help describe the functional response of microbial communities to potential environmental perturbations introduced by Marcellus shale activities. This study highlighted the potential impacts that fracking can have on headwater stream microbial communities and suggests that additional environmental studies are warranted to more fully characterize and integrate the potential environmental impacts at all trophic levels in these ecosystems.

### Conflict of interest statement

The authors declare that the research was conducted in the absence of any commercial or financial relationships that could be construed as a potential conflict of interest.

## Footnotes

^1^Pennsylvania is the Fastest-Growing Natural Gas-Producing State (2013).

^2^Pennsylvania Drives Northeast Natural Gas Production Growth (2011). Available online at: http://www.eia.gov/todayinenergy/detail.cfm?id=2870.
