# Supplementary material for: Assessing impacts of unconventional natural gas extraction on microbial communities in headwater stream ecosystems in Northwestern Pennsylvania
Source: Front Microbiol. 2014 Nov 4;5:522. doi: 10.3389/fmicb.2014.00522 (PMC4219493; doi:10.3389/fmicb.2014.00522)
Supplement: Supplementary file 1 [file DataSheet1.DOCX]

**Supplementary Information**

*Site Selection and Characteristics.* As described in Grant et al. stream selection targeted forested and remotely located watersheds within the Marcellus shale basin in northwestern Pennsylvania that contained naturally reproducing wild brook trout populations. Sites were first identified by utilizing GIS to overlay designated wild trout stream layer with a constructed unconventional shale gas permit layer. All watersheds were otherwise minimally disturbed with no evidence of mining legacy, and with conventional oil wells and dirt roads being the only other observed within watershed anthropogenic disturbances. All sites were sampled under baseflow conditions in June and July 2012.

All watershed features in this study are not significantly different to one another (p-values > 0.05). A number of land use metrics (e.g. wetlands, % forested, % coniferous, number of conventional oil wells), physical stream attributes (e.g. stream slope, stream length, drainage density), and basin characteristics (e.g. geology, watershed size, elevation) were compared between MSA+ and MSA- streams in this study and encompasses 24/26 sites analyzed. To our knowledge, 11 study sites were sampled prior to any well drilling or infrastructure construction and were located in pristine watersheds which exhibited no, or very minimal, prior anthropogenic activity. Fifteen sites were sampled after either some development (including road/pipeline/wellpad construction) or after complete, hydraulically fractured wells were present within the watershed. The number of wellpads/watershed in our dataset ranged from 0-11 (Overall mean=1.88; MSA+ mean=3.27) and the number of drilled wells/watershed ranged from 0-26 (Overall mean=4.38; MSA+ mean=7.60).

*DNA Extractions.* Samples were applied to Lysing Matrix E (LME) tubes (MP Biomedicals Santa Ana, CA) and 1 mL of CTAB extraction buffer, 500 µL of 25:24:1 phenol:chloroform:isoamyl alcohol, and 50 µL of ammonium aluminum sulfate was added. Subsequently, LME tubes were bead-beated using the DisruptorGenie (Scientific Industries, Bohemia, NY) at max speed for 2 minutes. Samples were spun at 16,000 x g for 10 minutes at 4˚C and the aqueous layer was removed and applied to 500 µL of chloroform. With the exception of water samples, LME tubes were washed again with CTAB solution, disrupted, and spun according to the aforementioned methodology. After application to pure chloroform, samples were vortexed vigorously for 10-15 seconds and spun at 16,000 x g for 5 minutes. The aqueous layer was removed and pipetted into a 30% polyethylene glycol solution and precipitated for 1 hour at room temperature. Once incubated, the samples were pelleted at 16,000 x g for 10 minutes. The supernatant was decanted and the pellet was washed with cold 70% ethanol and spun again at 16,000 x g for 5 minutes at 4˚C. The ethanol was aspirated from the pellet and excess ethanol was dried off at room temperature for no more than 5 minutes. The pellet was resuspended in Buffer EB (QIAGEN, Germantown, MD) and the DNA was then subjected to the AllPrep DNA/RNA Mini Kit (QIAGEN, Germantown, MD) using the manufacturers recommended protocol. Resulting DNA samples were quantified using the Qubit 2.0 flourometer (Life Technologies, Carlsbad, CA) and stored at -80˚ C.

*Library Purification, Verification, and Sequencing*

Duplicate PCR reactions were combined and purified using the Agencourt AMPure XP PCR Purification clean up kit (Beckman-Coulter Inc., Brea, CA) according to the manufacturer’s instructions. Clean PCR products were quantified using the Qubit 2.0 Fluorometer (Life Technologies, Carlsbad, CA), and samples were pooled in equimolar amounts. Prior to submission for sequencing, libraries were quality checked using the 2100 Bioanalyzer high sensitivity DNA chip (Agilent Technologies, Santa Clara, CA). Pooled libraries were stored at -20˚C until they were shipped on dry ice to the Children’s Hospital DNA Sequencing Core (Cincinnati, OH) for sequencing.

Library pools were size verified using the Fragment Analyzer CE (Advanced Analytical Technologies Inc., Ames IA) and quantified using the Qubit high sensitivity dsDNA kit (Life Technologies, Carlsbad, CA). After dilution to a final concentration of 1nM and a 10% spike of PhiX V3 library (Illumina, San Diego CA), pools were denatured for 5 minutes in an equal volume of 0.1N NaOH then further diluted to 12 pM in Illumina’s HT1 buffer. The denatured and PhiX-spiked 12 pM pool was loaded on an Illumina MiSeq V2 500 cycle kit cassette with 16S rRNA library sequencing primers [58] and set for 251 base, paired-end reads.

*Bioinformatics and Statistical Analyses*

Visualization of trends in microbial community structure for MSA+ and MSA- samples were generated in R using the *Phyloseq* package version 1.9.9. An unrarified OTU table and the corresponding .tre file and metadata table were merged into a phyloseq object. Taxa that were not observed in a count of 20 sequences or greater within any of the samples were removed using the ­function ‘filter_taxa.’ Remaining taxa were grouped at the phylum level using the function ‘tax_glom’ and further condensed using the function ‘tip_glom’ before plotting using ‘plot_bar’.

Alpha diversity rarefaction curves were generated within the QIIME-1.8.0 sequence analysis package using an OTU table that did not undergo single rarefaction, in an effort to maintain as much sequence data as possible. To generate alpha diversity rarefaction curves multiple rarefactions were conducted on sequences across all samples from minimum depth of 200 sequences, to a maximum depth of 11,200 sequences, with a step size of 1000 sequences/sample for 20 iterations. Alpha rarefactions were then collated and plotted using Phylogenetic Distance (PD) Whole Tree, Heip’s Evenness, Chao1 and observed species richness metrics. Alpha diversity was compared between MSA+ and MSA- communities using an unrarified OTU table at each taxonomic rank. Each summarized OTU table of each taxonomic rank underwent the same multiple rarefaction conditions previously described, which were collated using Heip’s Evenness, Chao1 and observed species richness metrics. Alpha diversity comparisons were then conducted using a two sample t-test and non parametric Monte Carlo permutations (n = 999) to yield p-values on each summarized OTU table’s respective collated file.

Variable sequencing depth was normalized across samples, by performing a single rarefaction at a depth of 11,208 sequences per sample, as this was the lowest number of sequences retrieved from a sample. The rarified OTU table was also filtered to remove singletons. Beta diversity was calculated using the weighted UniFrac distance metric and visualized with Principle Coordinates Analysis (PCoA) in EMPeror [65]. Analysis of Similarity (ANOSIM) was used to test differences in community structure within the samples (1000 Monte Carlo permutations). Adonis tests were performed on weighted UniFrac values to determine the amount of variation explained by stream water chemistry or watershed characteristics. Alpha levels of 0.05 were used to detect significance of categorical or continuous variables.

**Supplementary Figures**


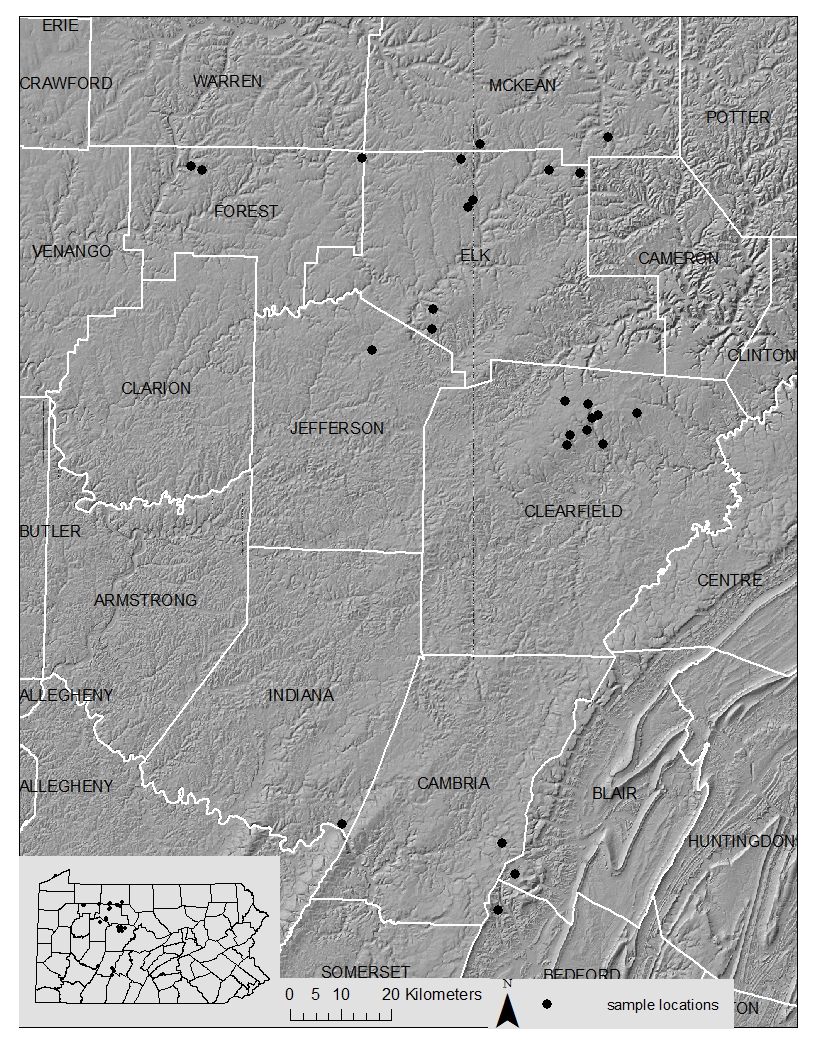


**Figure S1.** Stream sampling locations are shown using ArcGIS 10.0 to calculate watersheds, using a 1/3 arcsecond (approximately 10 m) resolution National Elevation Dataset, digital elevation model (DEM), and GPS coordinates for each sampling point.


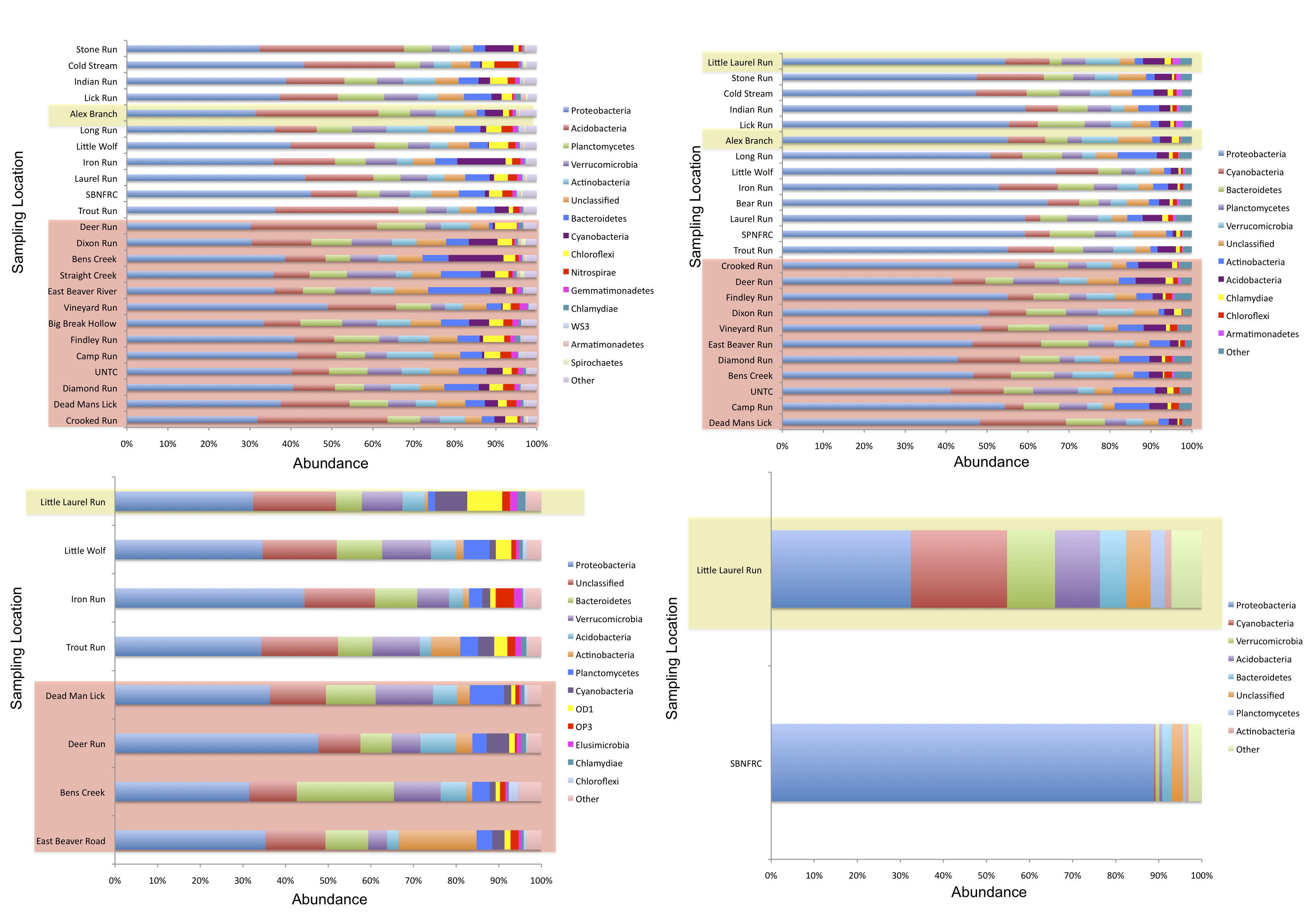


A B

`

C D

**Figure S2.** Relative abundance bar charts of OTUs at the phylum taxonomic rank within A) sediment, B) bryophyte, C) water and D) biofilm matrices. Relative abundance outputs were generated from an unrarified OTU table picked using the USEARCH sequence analysis tool. The y-axis displays all sampling locations within each sample matrix, and the x-axis displays phyla abundance. Phyla that constituted less than 1% of total sample community composition across all sample locations within each sample matrix were grouped together into the ‘other’ category. OTUs that QIIME could not identify at the kingdom level are grouped into the ‘unclassified’ category. Samples on the Y axis are arranged by decreasing number of wellpads from top to bottom, ending with samples boxed in red, all of which do not possess any wells. Samples highlighted in yellow have experienced documented fracking spills.


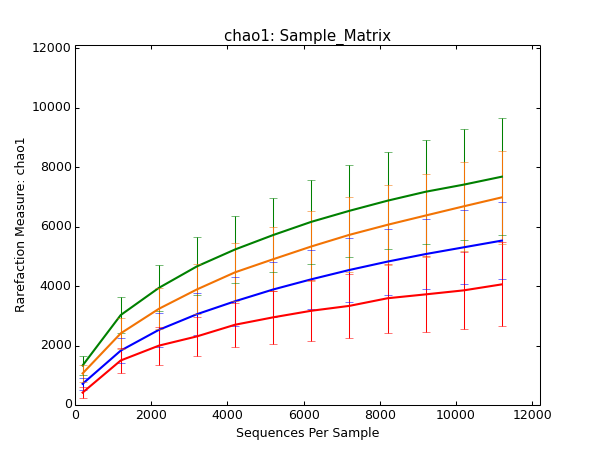

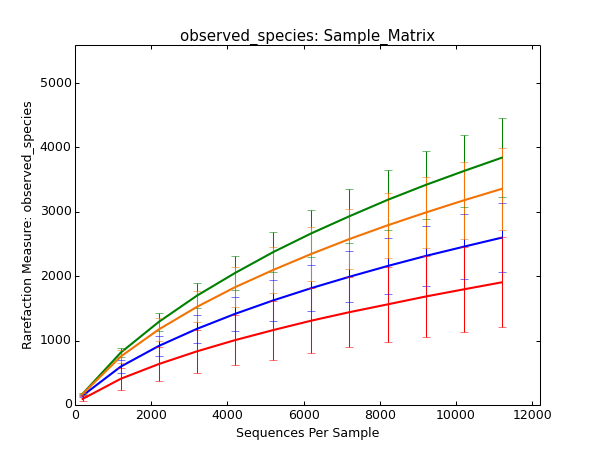


A B

**Figure S3.**  Alpha diversity rarefaction curves display differences in species richness between sediment, water, bryophyte and biofilm samples. Rarefaction curves were generated from an unrarified OTU table generated in QIIME 1.8.0. Multiple rarefactions were conducted on sequences across to a maximum depth of 11,200 sequences, with a step size of 1000 sequences/sample and 20 iterations. Alpha rarefactions were then collated and plotted using A) Observed Species, B) Chao1 metrics. Rarefaction curves present sequencing depth on the x-axis, and the number of unique species on the y-axis. Water samples (green) appear to have the greatest species richness, followed by sediment (orange), bryophyte (blue) and biofilm (red) samples.


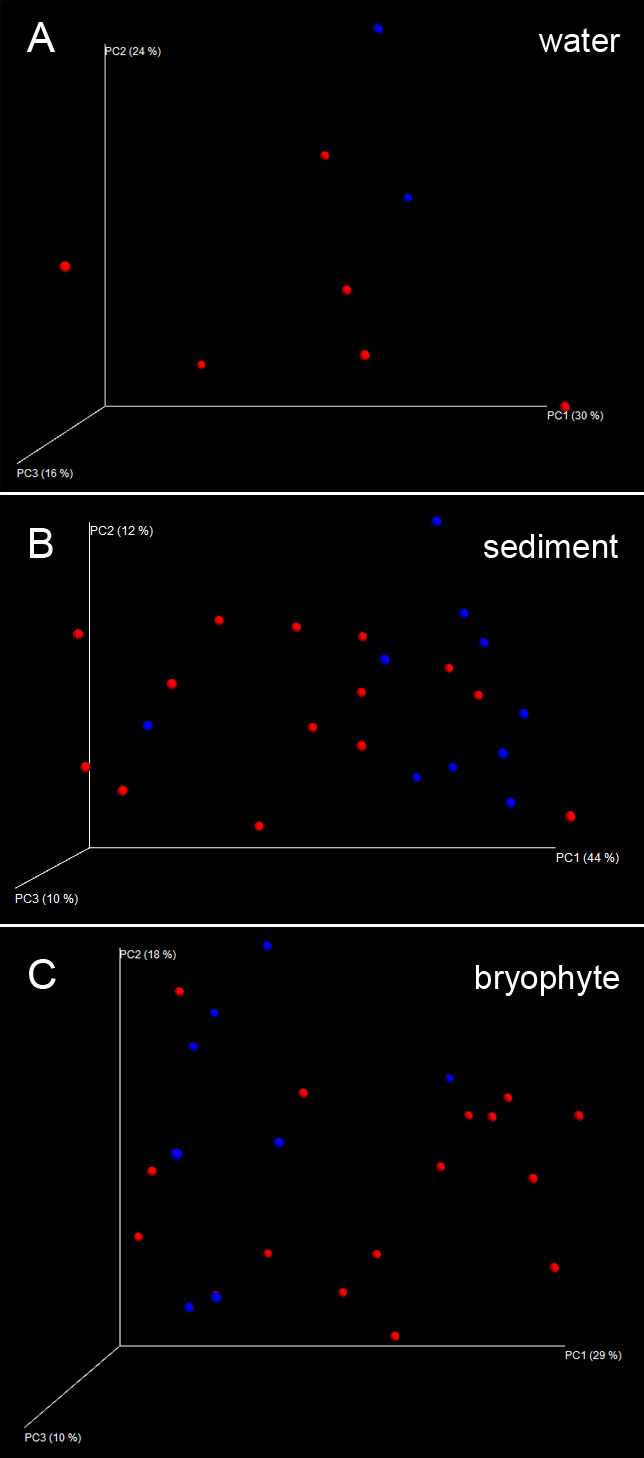


**Figure S4.** Principal Coordinates Analysis (PCoA) plots were used to visualize differences in weighted Unifrac distances of MSA+ (red) and MSA- (blue) samples within each matrix. Points clustered more closely together are more similar in terms of phylogenetic similarity, whereas points that are distant from each other are phylogenetically distinct. A) Communities sampled from stream water also tend to cluster by fracking status; however, small sample size limits the certainty of the trend (MSA+ n=6, MSA- n=2; ANOSIM p=0.338). B) Sediment communities cluster significantly by fracking status (MSA+ n=14, MSA- n=10; ANOSIM p=0.038). C) Bryophyte-associated communities also cluster fracking status (MSA+ n = 16, MSA- n = 8; ANOSIM p = 0.016).


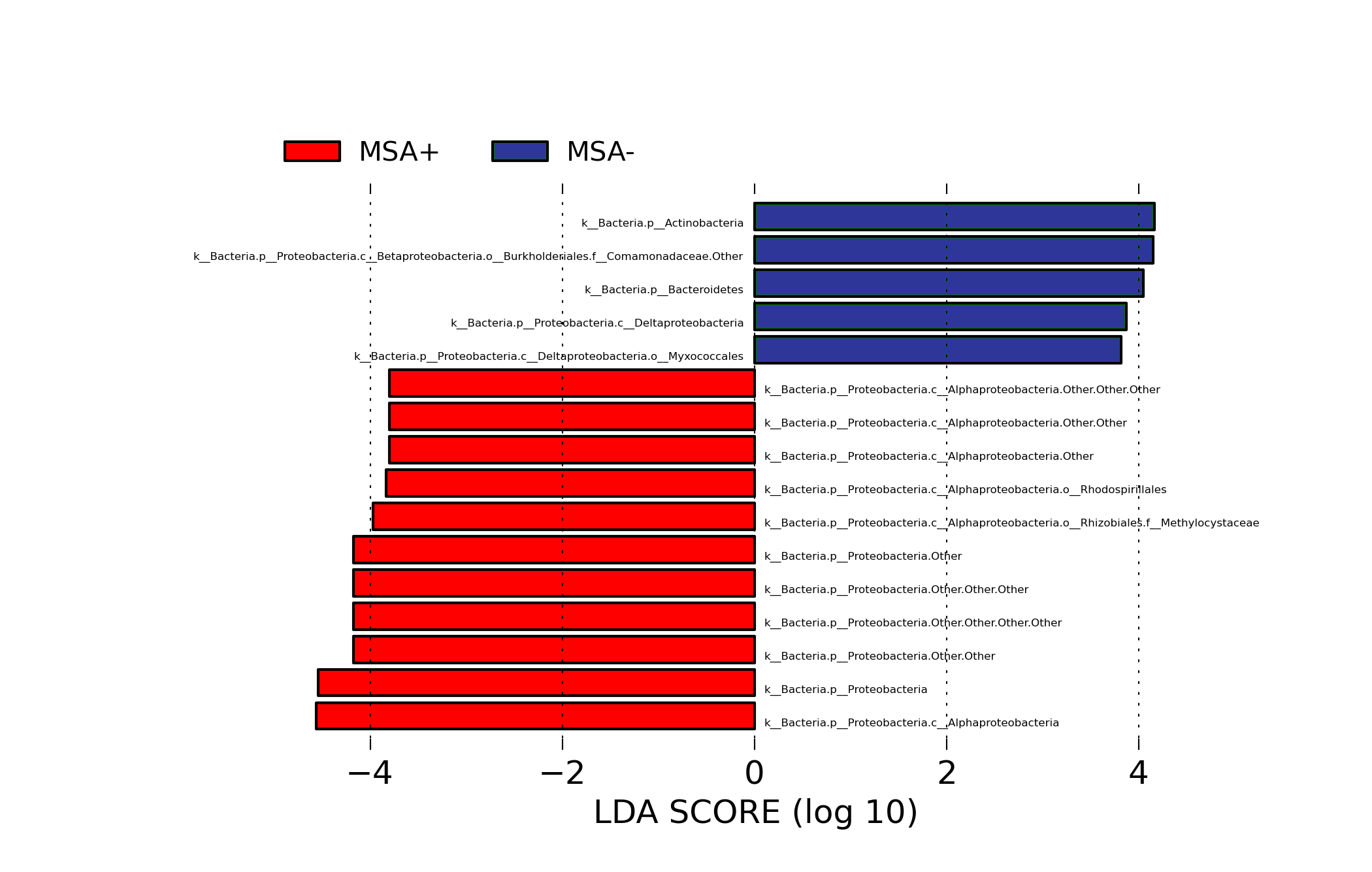


**Figure S5.** LEfSe plot of “biomarker” taxa using bryophyte-associated samples only (MSA+ n=15; MSA- n=9). The Kruskal-Wallis tests(α≤0.05) was used to determine significantly differentiating taxa between MSA+ and MSA- groups. Subsequently, a Linear Discriminant Analysis (LDA) was used to determine the effect size of taxa, which were then plotted on a log scale. Taxa in red were enriched in MSA+ water samples, while blue taxa are enriched in MSA- samples.


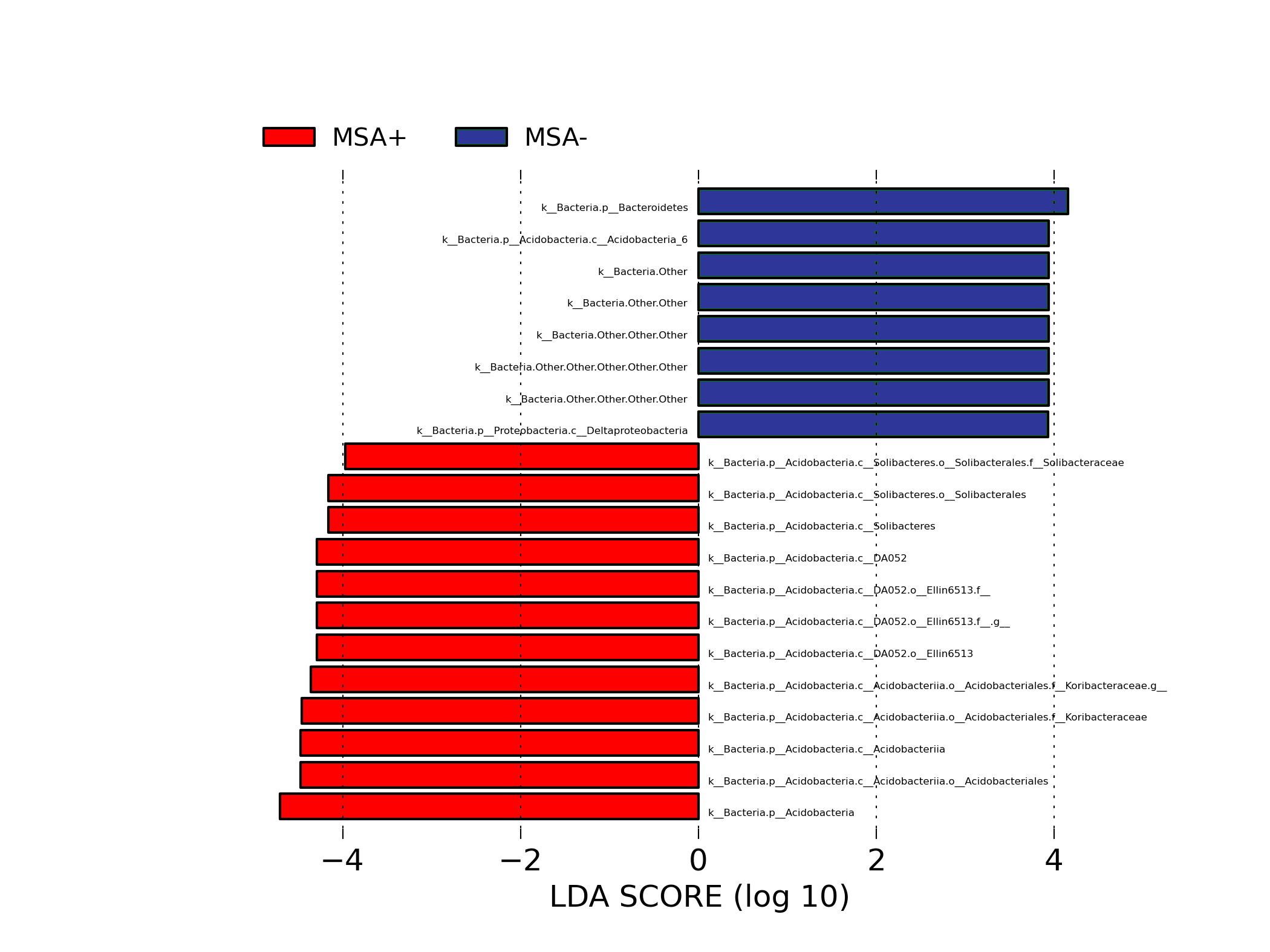


**Figure S6**. LEfSe plot of “biomarker” taxa using sediment samples only (MSA+ n=13; MSA- n=11). The Kruskal-Wallis test (α≤0.05) was used to determine significantly differentiating taxa between MSA+ and MSA- groups. Subsequently, a Linear Discriminant Analysis (LDA) was used to determine the effect size of taxa, which were then plotted on a log scale. Taxa in red were enriched in MSA+ water samples, while blue taxa are enriched in MSA- samples.


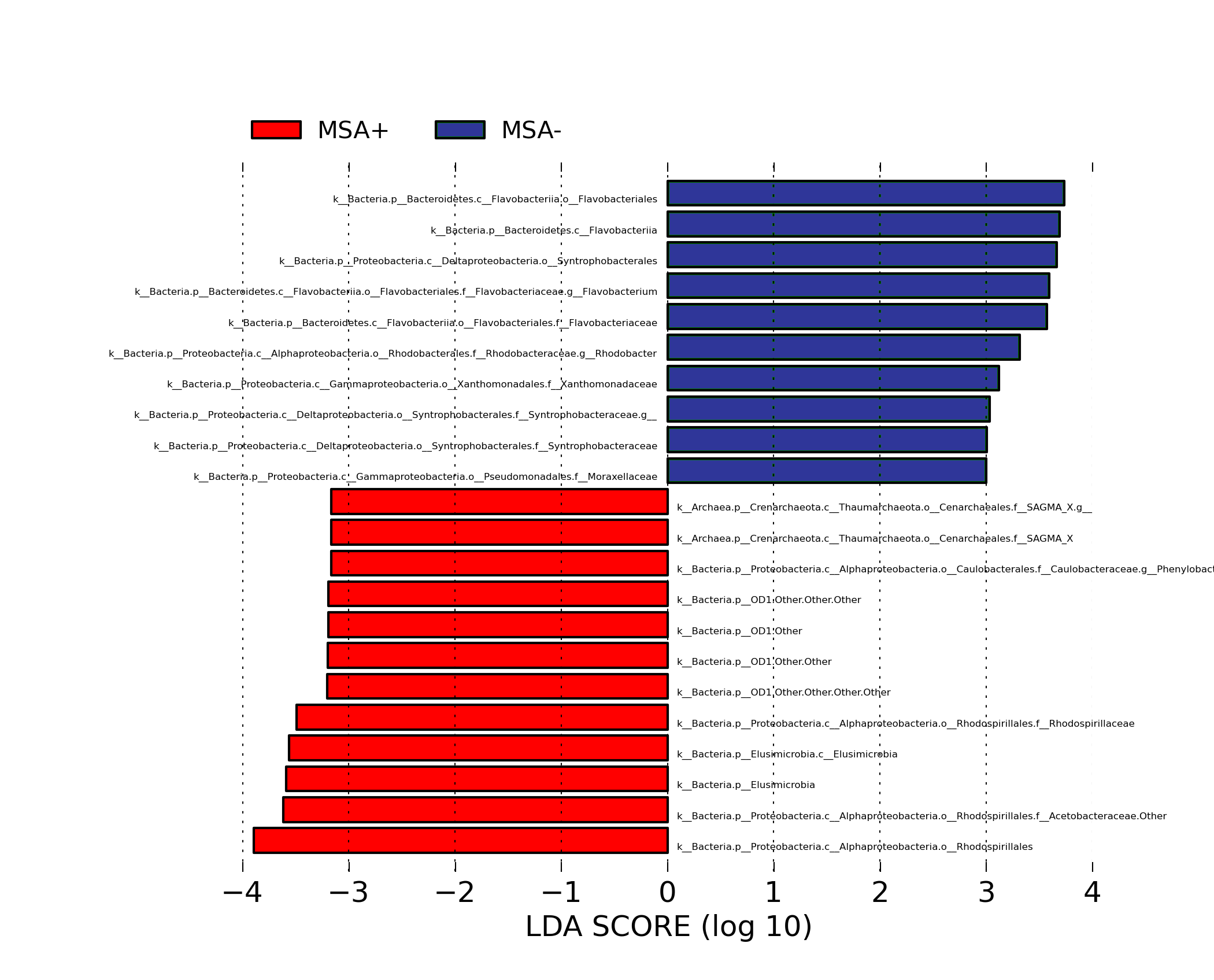


**Figure S7**. LEfSe plot of “biomarker” taxa using water samples only (MSA+ n=5; MSA- n=3). The Kruskal-Wallis tests (α≤0.05) was used to determine significantly differentiating taxa between MSA+ and MSA- groups. Subsequently, a Linear Discriminant Analysis (LDA) was used to determine the effect size of taxa, which were then plotted on a log scale. Taxa in red were enriched in MSA+ water samples, while blue taxa are enriched in MSA- samples.


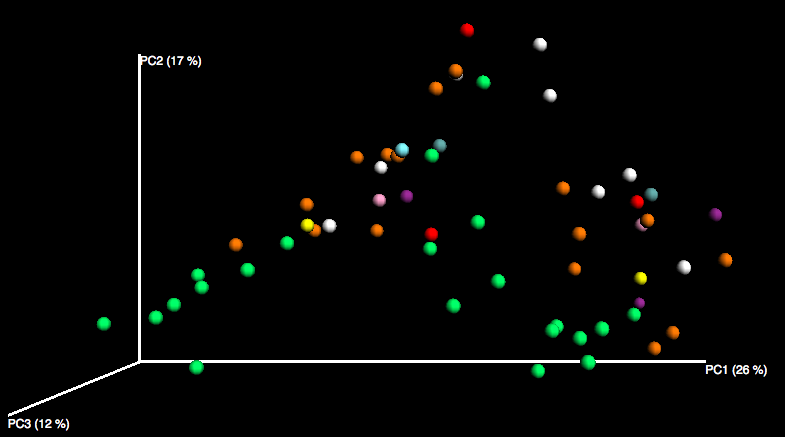


**Figure S8.** Principal Coordinates Analysis (PCoA) plot generated from a rarified OTU table of weighted UniFrac distances with all samples colored by county. Distinct clustering can be observed within samples collected from Clearfield county (light green) as well as some clustering between samples collected from Elk county (orange). The counties that were not affected by spill sites did not display clustering.

**Supplementary Tables**

**Table S1**. Spearman’s rho values reflecting the top ten most positive and most negative correlations of pH and Wellpads to bacterial genera.

|  | **pH** | **Wellpads** | **Total Nitrogen** |
| --- | --- | --- | --- |
| Niabella | 0.734307 | -0.61946 | 0.708005 |
| Flavobacterium | 0.721526 | -0.61467 | 0.682275 |
| Unc.^*^ Saprospiraceae | 0.695143 | -0.54511 | 0.746903 |
| Unc. Alteromonadales | 0.678829 | -0.65481 | 0.59436 |
| Unc. Acidobacteria-6 | 0.649296 | -0.65831 | 0.589073 |
| Rhodobacter | 0.639799 | -0.59088 | 0.685426 |
| Unc. Pedosphaerales | 0.639482 | -0.5868 | 0.764205 |
| Unc. Betaproteobacteria | 0.637235 | -0.63461 | 0.647798 |
| Unc. Myxococcales | 0.62926 | -0.60572 | 0.591866 |
| Unc. Anaerolineae | 0.626355 | -0.58518 | 0.640436 |
| Unc. Methylocystaceae | -0.74626 | 0.546127 | -0.71448 |
| Unc. Armatimonadia | -0.63246 | 0.513174 | -0.55575 |
| Phenylobacterium | -0.62144 | 0.441219 | -0.56448 |
| Unc. Acidobacteriales | -0.61983 | 0.408077 | -0.64144 |
| Unc. Acetobacteraceae | -0.61098 | 0.552614 | -0.57521 |
| Unc. Acetobacteraceae | -0.61058 | 0.567824 | -0.63269 |
| Unc. Sphingobacteriaceae | -0.60768 | 0.313714 | -0.59957 |
| Candidatus Solibacter | -0.5997 | 0.47705 | -0.48067 |
| Unc. Chthonomonadaceae | -0.58396 | 0.439987 | -0.62448 |
| Unc. Solibacteraceae | -0.58011 | 0.349854 | -0.44489 |
| Unc. Caulobacteraceae | -0.50412 | 0.556748 | -0.50331 |
| Unc. Acidobacteriaceae | -0.57221 | 0.505943 | -0.50781 |
| Isosphaeraceae | -0.45451 | 0.467438 | -0.38218 |
| Telmatospirillum | -0.5609 | 0.44989 | -0.66467 |
| Devosia | 0.520289 | -0.66337 | 0.299823 |
| Xanthomonadaceae | 0.418513 | -0.62792 | 0.324588 |
| Unc. Bacteroidetes | 0.594183 | -0.61262 | 0.43929 |

*‘Unc.’ Denotes an unclassified taxa.

**Table S2. Pairwise** Bray-Curtis dissimilarities and unweighted unifrac distances between all pairs of counties.

|  | Metric | Cambria | Clearfield | Elk | Forrest | Jefferson | McKean | Bedford | Westmoreland |
| --- | --- | --- | --- | --- | --- | --- | --- | --- | --- |
| **Clearfield** | Bray-Curtis | 0.85 |  |  |  |  |  |  |  |
| **Elk** | Bray-Curtis | 0.74 | 0.59 |  |  |  |  |  |  |
| Forrest | Bray-Curtis | 0.64 | 0.77 | 0.52 |  |  |  |  |  |
| Jefferson | Bray-Curtis | 0.68 | 0.90 | 0.84 | 0.76 |  |  |  |  |
| McKean | Bray-Curtis | 0.56 | 0.87 | 0.80 | 0.73 | 0.66 |  |  |  |
| Bedford | Bray-Curtis | 0.74 | 0.95 | 0.89 | 0.82 | 0.83 | 0.73 |  |  |
| Westmoreland | Bray-Curtis | 0.54 | 0.89 | 0.81 | 0.73 | 0.70 | 0.56 | 0.68 |  |
| Blair | Bray-Curtis | 0.66 | 0.93 | 0.83 | 0.73 | 0.76 | 0.69 | 0.64 | 0.61 |
| Clearfield | UniFrac | 0.62 |  |  |  |  |  |  |  |
| Elk | UniFrac | 0.58 | 0.56 |  |  |  |  |  |  |
| Forrest | UniFrac | 0.56 | 0.61 | 0.49 |  |  |  |  |  |
| Jefferson | UniFrac | 0.57 | 0.68 | 0.66 | 0.62 |  |  |  |  |
| McKean | UniFrac | 0.57 | 0.66 | 0.66 | 0.66 | 0.56 |  |  |  |
| Bedford | UniFrac | 0.65 | 0.78 | 0.73 | 0.71 | 0.66 | 0.63 |  |  |
| Westmoreland | UniFrac | 0.55 | 0.68 | 0.64 | 0.63 | 0.58 | 0.56 | 0.59 |  |
| Blair | UniFrac | 0.61 | 0.74 | 0.66 | 0.64 | 0.62 | 0.62 | 0.58 | 0.57 |
